# Innovations in air sampling to detect plant pathogens

**DOI:** 10.1111/aab.12191

**Published:** 2015-01-19

**Authors:** JS West, RBE Kimber

**Affiliations:** 1 Department of Plant Biology and Crop Science, Rothamsted ResearchSt Albans, UK; 2 South Australian Research and Development Institute (SARDI)Adelaide, South Australia, Australia

**Keywords:** Aerobiology, cyclone, diagnostics, GIS, IDM, IPM, UAV, virtual impactor

## Abstract

Many innovations in the development and use of air sampling devices have occurred in plant pathology since the first description of the Hirst spore trap. These include improvements in capture efficiency at relatively high air-volume collection rates, methods to enhance the ease of sample processing with downstream diagnostic methods and even full automation of sampling, diagnosis and wireless reporting of results. Other innovations have been to mount air samplers on mobile platforms such as UAVs and ground vehicles to allow sampling at different altitudes and locations in a short space of time to identify potential sources and population structure. Geographical Information Systems and the application to a network of samplers can allow a greater prediction of airborne inoculum and dispersal dynamics. This field of technology is now developing quickly as novel diagnostic methods allow increasingly rapid and accurate quantifications of airborne species and genetic traits. Sampling and interpretation of results, particularly action-thresholds, is improved by understanding components of air dispersal and dilution processes and can add greater precision in the application of crop protection products as part of integrated pest and disease management decisions. The applications of air samplers are likely to increase, with much greater adoption by growers or industry support workers to aid in crop protection decisions. The same devices are likely to improve information available for detection of allergens causing hay fever and asthma or provide valuable metadata for regional plant disease dynamics.

## Introduction

Dispersal in air is one of many mechanisms by which plant pathogens can spread to reach new susceptible plants either within the same field or even in a completely different continent (Pady & Kapica, 1955; Gregory, 1973; Brown & Hovmøller, 2002). Gregory (1973) predicted that most spores of plant pathogens do not disperse beyond the field in which they were produced. Although this short-distance dispersal can be important for disease epidemics by infection of new plants in the same field or adjacent fields around inoculum sources, longer distance dispersal is also of great significance in allowing spores to reach new locations. In some cases, it is possible for pathogens to travel to new continents, surviving extremes of cold temperature, UV light and desiccation to remain viable and able to cause disease (Morris *et al.*, 2013; Gregory, 1952). These include bacteria, fungi and viruses that can be vectored in pollen (Shiller *et al.*, 2010).

The Hirst spore trap (Hirst, 1952) has become the workhorse of aerobiological sampling for over 60 years. The simplicity of its design has been a key reason for this – comprising just three moving parts: electric motor, bearing to turn the air intake into the wind, and a clockwork mechanism to move the collection surface past the air intake. It has been used in hundreds of studies of airborne pollen and plant- or fungal-spores, particularly in modified forms such as the Burkard seven-day spore trap, which replaces collection onto a glass slide by collection of particles onto adhesive tape on a circular drum that rotates over a period of seven days. This has provided valuable information in the fields of plant pathology and the monitoring of pollen and other airborne allergens. This review discusses a range of more recent developments in air sampling devices that could be used in plant pathology. Many of these have been developed to improve the ease of use for processing captured samples, to present captured air-particulate samples in new formats compatible with rapid application of diagnostic methods. In some recent examples, developments include full-automation of all steps from sample collection to detection of target organisms or genetic traits and wireless reporting of the result.

This review will concentrate on detection of specific biological particles. The main focus of this review will be directed at developments applicable to plant pathology, which was the original purpose of the Hirst trap, and which allows identification or quantification of individual particles, such as a species of a fungus. These developments include the MTIST, miniature cyclone, improvements to the rotorod and passive traps, the Jet spore trap, Coriolis trap, ionic spore trap, liquid impingers, wet-walled cyclones, and the Miniature Virtual Impactor. The review concentrates on developments of air samplers and does not include methods to monitor rainfall for such pathogens. Monitoring rain is receiving great interest because species such as *Pseudomonas syringae* may be present in air in sufficient concentrations to affect the hydrological cycle, by ice nucleation activity to induce rainfall to aid their dispersal. Rain sampling methods have been developed to sample for long-distance transported plant pathogens such as those causing cereal and soybean rusts (Barnes *et al.*, 2006; http://www.ars.usda.gov/Main/docs.htm?docid=14549; King (2014)). It has also been suggested that some splash-dispersed pathogens can be dispersed significant distances because of effects of strong wind and rain (Gottwald *et al.*, 2002; Perryman *et al.*, 2014).

Although the Hirst trap was devised for analysis of the sample using light microscopy, for some target organisms, accurate diagnosis by visual assessment is impossible because of spores of many other species having the same appearance. Microscopy is still used and is a relatively easy method for identification of distinctive spores and pollen. High throughput analysis using microscopy has been assisted by use of automated image-recognition techniques (e.g. http://www.aeromedi.org/). However, a driving force in development of recent air samplers has been consideration to post-sampling techniques applied to add precision to identification of particles by use of techniques such as: immunological methods (e.g. ELISA, lateral flow devices), DNA-based diagnostics (e.g. PCR, TwistDX and LAMP), and biosensors (e.g. the SYield autosampler, which wirelessly reports results on-site without the sample being taken to a lab). For a full review of available diagnostic methods, see Heard & West (2014).

Aerobiological sampling using mobile platforms, such as Unmanned Aerial Vehicles (UAVs), is a rapidly evolving area of research that combines the science of microbiology with the advances in engineering and technology. This has added a new complication to interpreting results of an ever increasing use of air sampling in plant pathology because to interpret results of this technology, care is needed to understand the relationship between actual exposure of crops to airborne inoculum, the air-sampler type (whether an active volumetric or passive trap, their collection efficiency and the volume of air sampled per minute) and the sampler location (proximity to crops and height above ground). Thresholds of airborne spores indicating disease risk, vary greatly depending on the location of the sampler and infectivity or viability of the spores. Monitoring systems may be improved by targeting periods of risk indicated by air-trajectory analysis, knowledge of likely sources, the effect of weather on the biology of spore release and the density of susceptible crops, which can be indicated by remote sensing. Clearly, integration with precise diagnostic methods will continue to provide new information about the epidemiology of pathogens and also the occurrence of genetic traits such as fungicide resistance and new pathotypes (Fraaije *et al.*, 2005; Kaczmarek *et al.*, 2014).

## Air samplers

Airborne particulates can be sampled in a number of different ways. These have different advantages and disadvantages in aspects of air sample flow rate, collection efficiency (which usually varies with particle size), power consumption, length of sample period, length of period they can be left unattended and ease of processing the samples.

The simplest devices work by passive deposition or passive impaction of particles onto adhesive surfaces such as a petroleum-jelly (Vaseline)-coated microscope slide or Vaseline-coated thin glass rod). This method can be useful for comparative purposes and as a relatively cheap method but does not allow a calculation of the concentration of spores in air. Nevertheless, for monitoring purposes, this has proven to be an effective method and is particularly useful in remote locations where power supply could pose a problem. These devices are still used in monitoring plant pathogen spores – e.g. for monitoring coffee rust and sugarcane rust in Mexico, a device comprising a Vaseline-coated microscope slide, held at 45° inside a short length of plastic drain-pipe has been used (pers. com. Carmen Calderón Ezquerro, UNAM, Mexico). The drain-pipe acts as a rain-shield and the device can turn into the wind by being mounted on a bearing and having a wind vane at the back. Similar devices have been used in the USA for monitoring various rust fungi such as soybean rust (von Qualen & Yang, 2006).

Actively sampled volumetric methods are more routinely used as these allow a calculation of particles per m^3^ of air. Volumetric methods include collection by impaction (various methods – see Table1 below), impinging (SKC biosampler, Zefon Fritted Midget Impinger), filtering (Button, IOM), virtual impaction (MVI), cyclone (dry, wet-walled and two-stage) and electrostatic attraction (Ionic) (Cox & Wathes, 1995; Lacey & West, 2006; Jackson & Bayliss, 2011). Impaction methods can include impaction onto solid surfaces, such as agar in a Petri-dish (Andersen sampler, SKC Biostage and many others), or adhesive-coated tape, slides or rods (Hirst-type spore trap, Burkard seven-day spore trap, Air-O-Cell, rotorod and rotating-arm spore trap), or it can be impacted onto a liquid (wet-walled cyclone, Coriolis, CIP 10-M) (Table1). Choice of sampler depends on power availability (mains or battery and/or solar panel), optimal format for analysis or automated detection, volume of air sampled per minute and duration of sampling. The Hirst, Burkard seven-day and some impactors that rotate the agar-plate beneath an air-intake slit, have the added advantage of informing at what time of the day, certain spores were present in the air, which can be associated with various environmental (rain, wind, temperature or humidity effects) or operational variables (mechanical or human-mediated operations). The optimal format may include culturing, in which case an important consideration is maintaining viability of spores collected. Viability can be reduced by effects of impaction at high speeds, desiccation and chemicals. The air volume sampled is important, with higher volumes needed for the detection of relatively rare spores but often there is a trade-off against sample period and energy use because some devices can only work for minutes or hours rather than weeks because of collection surfaces becoming overloaded, collection liquid evaporating or batteries losing power. One impressive system developed for sampling over long periods (a week or more) at high volumes (900 L min^−1^) is the ChemVol High Volume Cascade Impactor (Buters *et al.*, 2012; Table1). For collection of very small particles, particularly below 1 µm aerodynamic diameter, wet cyclones or liquid impingers are the best samplers to use – the Coriolis Air Samplers can operate at 100–600 L min^−1^ for up to 6 h if circulating fluid is replenished periodically. Wet-walled cyclones and liquid impingers that use swirling liquid and the CIP 10-M (Table2) have an additional advantage of maintaining viability of cells that can be killed by impaction onto solid surfaces. For example, the SKC biosampler directs airflow via three tangential nozzles to create a swirling airflow that causes a liquid collection medium to swirl along the inside surface of the glass collection chamber, causing nearly 100% collection efficiency for particles over 1 µm and 90% efficiency for 0.5 µm (aerodynamic diameter) particles (Willeke *et al.*, 1998). The CIP 10-M sampler has horizontal blades on the upper part of a rotating cup, filled with collection liquid, to generate a helical airflow to place living cells gently on the liquid-film to maintain viability. Loss of viability by impaction onto a solid surface is reduced in the ChemVol High Volume Cascade Impactor (Table1), which uses a polyurethane foam as the solid impaction surface because the high loading capacity of this material is thought to reduce impaction stress. Another method that maintains viability, completely avoids impaction onto a solid surface at high speeds by ‘virtual impaction’, where air is drawn through an accelerator ‘jet’ into a collection chamber of still air, which causes the air flow to change direction to exit the still chamber, with particles separating from the air flow because of their momentum, to settle passively in the collection chamber (Lacey & West, 2006). Virtual impactors can operate at high flow rates because of lack of restrictions such as filters, for example, the Burkard High Volume Jet spore trap (Limpert *et al.*, 1999), samples at 850 L min^−1^. A miniaturised version, the MVI operates at 20 L min^−1^ (West *et al.*, 2013).

**Table 1:** Descriptions of commercially available spore traps based on impaction of particles for collection

Sampler	Description	References	Flow Rate (L min^-1^)	Collection Efficiency d50 (µm)^a^	Sample period	Compatible with analysis method		
						Culture	Microscope	Biosensor/molecular
Burkard Seven Day http://www.burkard.co.uk/7dayst.htm	Impaction (Hirst-type with rotating collection surface)	Lacey & West (2006)	10	2.2	7 days	No	Yes	Y – requires processing steps
Andersen sampler	Multi-stage (cascade) impactor onto agar plates via progressively finer air inlet-pores to fractionate particles by size	Andersen (1958)	28.6	Stage 1 = 8 Stage 6 = >0.43	0.2–20 min otherwise collection plates are overloaded	Yes	Processing needed to make a spore suspension or collect onto waxed slides	Yes - requires processing steps
Marple Series 290 Personal Cascade Impactor http://www.newstarenvironmental.com/product/marple-series-290-personal-cascade-impactor	A miniaturised multi-stage personal cascade impactor with terminal filter	Rubow *et al.* (1987)	2	0.52-21.3 (in stages)	<1 day	Yes – after processing	Yes	Yes – after processing
Airtrace environmental http://www.pmeasuring.com/particleCounter/microbialAirSamplers/AirTrace	Impaction via air intake slit onto agar-plate		28.3		1 hour	Yes	Processing needed to make a spore suspension	Yes – after processing
Rotorod or rotating-arm (various manufacturers)	Impaction onto leading edge of rotating-arms	Lacey & West (2006)	Typically 100-150	10-20	Typically up to 12–24h before overloaded depending on concentration	No	Yes	Yes – requires processing steps
Air-O-Cell http://www.zefon.com/store/air-o-cell-sampling-cassette.html	Impaction onto adhesive-coated slide	Kalatoor *et al.* (1995)	4	<1		Yes (suspension and plating needed)	Yes	Yes – requires processing steps
ChemVol High Volume Cascade Impactor http://o.b5z.net/i/u/10004484/i/brochures/Particulate%20Monitoring/ChemVol_Model_2400_Brochure.pdf	Four, progressively finer impaction stages and a terminal ultra-fine filter. The poly urethane foam impaction substrate reduces particle bounce and allows sampling over extended periods	Buters *et al.* (2012)	900	Large >9 Coarse = 9-4 Fine = 4-1 Forth = 1-0.2 Ultrafine <0.08	>1 week	Yes (suspension and plating needed)	Yes (suspension needed)	Yes
MTIST	Impaction (into microtitre-plate wells)	Kennedy *et al.* (2000)	57	≈2	Up to 7 days	Yes	Yes (suspension in liquid needed)	Yes

a d50 is the aerodynamic diameter or size of particles above which 50% or more are collected and below which, less than 50% are collected.

**Table:** Descriptions of cyclone and wet-walled cyclone spore traps

Sampler	Description	Ref	Flow rate (L min^-1^)	Collection Efficiency d50 (µm)*	Sample period	Compatible with analysis method		
						Culture	Microscope	Biosensor/molecular
Burkard cyclone/multivial cyclone http://www.burkard.co.uk/autocycsamp.htm	Dry cyclone sampling into 1.5 mL tube into which particles settle because of a change in direction of vortexing air as it leaves via its central axis	González-Parrado *et al.* (2014) Bock & Cotty (2003)	16.6	20+		Yes (suspension and plating needed)	Yes (suspension in liquid needed)	Yes
NIOSH one-stage BC 112 sampler and NIOSH 2-stage BC 212 sampler	Dry cyclone or two-stage cyclone with terminal filter cassette (Various filters are compatible)	(1-stage): Chen *et al.* (2004).“. (2-stage) Lindsley *et al.* (2006)	4 (1-stage) 3.5 (2-stage)	1.5 (1-stage) 1.8 (2nd of-stage) and 1 (1^st^ of 2-stage)	Variable	Yes	Needs processing	Yes
Coriolis®μ and Coriolis Recon Air Samplers http://www.coriolis-airsampler.com	Wet-wall cyclone	Carvalho *et al.* (2008)	100-300 (μ) 600 (Recon)	<1	1-10 minutes (μ) Up to 6h with long term facility option	Yes	Yes	Yes
Biral Aerosol Particle Collector http://www.biral.com/apc	Wet-wall cyclone		‘high’	1	‘Many hours’ depending on replenishment of circulating fluid	Yes	Yes	Yes
SKC biosampler http://www.skcltd.com/index.php/air-sampling-pumps/9-uncategorised/208-biosampler	Wet-wall cyclone	Willeke *et al.* (1998)	12.6	<0.2	<8h	Yes	Yes	Yes
Biotrace Biological Detection System (BBDS)	Wet-walled cyclone		750	<1	Continuous	A continuous flow ATP detector utilises ATP bioluminescence, for rapid detection of viable microbes in air		
CIP 10-M Personal microbe sampler	gentle impaction onto a liquid-filled rotating cup	Görner *et al.* (2005); Nieguitsila *et al.* (2011)	10	1.8	1–200 min	Yes	Yes	Yes

* d50 is the aerodynamic diameter or size of particles above which 50% or more are collected and below which, less than 50% are collected.

The collection efficiency of samplers depends on the speed of the airflow through the air intake (or speed of rotation of rotating arms), the width of the air intake and the separation distance between the intake and the collecting medium (or width of rotating arms). Particles smaller than 1–2 µm can rapidly change direction with the airflow, while larger, heavier particles are carried forward by their momentum to impact onto the collection medium. For samples collected over a long period onto solid media such as adhesive tapes, part of the sample may be lost through re-suspension into the air-stream or ‘bounce-off’ when new particles hit other particles rather than adhesive medium. This problem can be solved by moving the adhesive collection surface past the air intake (e.g. Hirst or Burkard spore samplers), by changing to new collection vials (e.g. Burkard multi-vial cyclone) or by continuously washing collection surfaces with liquid (e.g. wet-cyclone samplers or liquid impingers). In all cases, collection media and/or handling conditions should aim to inhibit spore germination, which would produce over-estimates of spore numbers assessed by qPCR.

## Impactors

Impactors can have relatively high collection efficiencies above a certain ‘cut-off’ particle size and can also sample at relatively high volume rates. Many were designed for assessment by culturing or microscopy. However, the MicroTitre Immuno Spore Trap or MTIST also works by impaction of air directed through a series of trumpet-like passages opening into each well of a microtitre plate. The airborne particles become impacted into the wells, with collection efficiency enhanced by adding an adhesive coating such as albumin. This sample format was intended for direct application of immunological diagnostic methods, particularly ELISA. As the total air flow is collected into 32 separate wells, it is possible to perform separate tests for different target organisms (Kennedy *et al.*, 2000). Many impactors have been adapted for samples to be analysed by DNA-based diagnostics such as PCR (Calderon *et al.*, 2002; Carisse *et al.*, 2009a).

## Cyclones and wet-cyclones

Similarly to the MTIST, the miniature cyclone or multi-vial cyclone, was developed to simplify handling of the sample by sampling directly into an Eppendorf tube for easy application of immunological or DNA-based diagnostic methods rather than microscopy. The sampler operates at 16.6 L min^−1^ by vortexing air into a chamber above the collection tube, where particles are collected as they separate from the air flow. For example, Pashley *et al.* (2012) used a Burkard cyclone to sample air that for three selected contrasting days, samples were sequenced and compared to data produced by visual observations under a microscope. They found that over 86% of genera detected by sequencing were not routinely identifiable by microscopy. The multi-vial version comprises a cyclone sampler mounted on a carousel of eight collection tubes, which can be programmed to rotate a new collection tube into place for collection at a desired time. This level of automation and ease of processing is tempered by variable collection efficiency, gradual build-up of deposited particles in the body of the cyclone above the collection tube and a tendency for the sample tubes to fill with water in foggy conditions. Multi-vial cyclones are used in the Brassica Alert network of spore traps that provide a direct, inoculum-based warning of airborne spores (http://www.syngenta-crop.co.uk/brassica-alert). The network collects samples in different locations, which are tested using a lateral flow immunological assay for different pathogens, only when the weather conditions are suitable for infection. This makes the system relatively inexpensive and has reduced fungicide application to crops that would have occurred if farmers relied only on weather-based infection conditions to indicate disease risk. Lateral flow device tests applied to cyclone air samples have also been developed to provide warnings of *Peronospora destructor* (onion downy mildew) (Kennedy & Wakeham, 2008).

## Electrostatic impactors and impingers

Impingers bubble air through a liquid, which is good at collecting very small particles that would not be impacted with high efficiency because of them changing direction with the airflow. However, the air volume sampled per minute is usually relatively low and the choice of liquid used, depends on the miscibility of the particles under study – hydrophobic spores may require use of alcohol or water with a surfactant to encourage the spores into suspension. This type of sampler can only be operated for short periods unless the collection liquid is topped up to replace liquid lost by evaporation. For example, the May multistage liquid impinge (May, 1966), samples into three different liquid stages at 50, 20 or 10 L min^−1^ (depending on size of device used) with 50% collection efficiencies at 6 µm (stage 1), 3.3 µm (stage 2) and <2 µm (stage 3). The device can be operated typically for up to 45 min or longer if the collection liquid is replenished. Samples can be used for culturing and a wide range of molecular diagnostic methods.

A different approach, electrostatic attraction, was used by the ‘Ionic spore trap’ (Schneider *et al.*, 2007; http://ionicsporetrap.com/), which is able to sample at flow rates up to 660 L min^−1^ and for up to 2 days (or longer in very clean environments) by charging particles in a strong electric field as they enter the trap, they become attracted to an electrode that is an electron microscope stub. The particles that collect on the stub can be viewed by scanning electron microscope (SEM) or conventional diagnostic methods applied. Collection efficiency is high for particles over 2 µm diameter, while smaller particles are collected but efficiency depends on the speed of airflow past the collecting surface (Pers. comm. Prof. Ray W. Schneider, Louisiana State University Agricultural Center, Baton Rouge).

## Virtual impactors

Virtual impactors force a jet of air into a chamber where the airflow changes direction, causing particles that have greater momentum than air molecules, to separate from the main airflow. The separated particles comprise a ‘minor flow’ which can be enhanced to avoid rejoining the majority of airflow by a relatively small suction force being applied to encourage the separated particles to either settle passively or be impinged or impacted by a secondary trap process e.g. Patents US005498271A and US4301002 both use a virtual impactor with downstream filters to sample the minor and major flows of the virtual impactor. The ‘Jet spore trap’ is a type of virtual impactor that was used by Limpert *et al.* (1999) to sample large volumes of air (850 L min^−1^) into a settling chamber above Petri-dishes containing detached leaves of cereal plants. These were incubated to produce pustules on leaves that could then be sub-sampled. An innovative aspect in this study, was that the sampler was mounted on the roof of a car and driven at speeds that caused sufficient air-flow into the device by use of a venturi (see Section Sampler locations: field, rooftop and airborne platforms). The study showed that the diversity of pathotypes of barley powdery mildew increased as the sampling journey passed from the west of Europe (the UK) eastwards into continental Europe. Sample flow rates (Table3) vary from 20 L min^−1^ for the Miniature Virtual Impactor (West *et al.*, 2013) to 850 L min^−1^ for the Jet spore trap (Limpert *et al.*, 1999).

**Table 3:** Comparison of commercially available virtual impactors for air sampling

Sampler	Description	References	Flow Rate (L min^−1^)	Collection Efficiency d50 (µm)*	Sample period	Compatible with analysis method		
						Culture	Microscope	Biosensor/ Molecular
VeroTect bio-detector http://www.biral.com/bio-detectors/biodetectors	Virtual Impactor	Shelton *et al.* (2004)	33	0.5	Continual			
						Aerosol Size And Shape Characterisation and additional generic characterisation by particle fluorescence using 280 nm excitation can provide a simple yes/no indication of biological particles or a more sophisticated analysis package for research use.		
Biral Aspect http://www.biral.com/imagprod/downloads/Aspect.pdf	Virtual Impactor	Shelton *et al.* (2004)	?	?	Continual			
						Aspect uses light scattering to measure particle shape as well as size and so is suitable for small particles and high throughput rates than systems that rely on particle imaging.		
Burkard Jet spore sampler http://www.burkard.co.uk/jetsamp.htm	Virtual Impactor	Limpert *et al.* (1999)	850	1–2 (estimate)	Up to several days	Yes	Yes	Yes
Miniature Virtual Impactor (MVI)	Miniature Virtual Impactor	West *et al.* (2013)	20	2 (estimate)	Up to several days	Yes	Yes	Yes

* d50 is the aerodynamic diameter or size of particles above which 50% or more are collected and below which, less than 50% are collected.

## Filters

Filters usually have a low flow rate because of resistance caused by the filter, unless, as in the DFU 1000, the filter is designed to have a very large surface area (Table4). In some cases, this means that the collected sample is dispersed over a large filter, which can be avoided by use of virtual impactors to concentrate airborne particles into a chamber that is then sampled either by passive deposition or a more active process such as filtering or impinging the minor flow of the virtual impactor (see above). Alternatively, there may be pre-separation stages, such as cyclones ahead of a terminal filter (e.g. NIOSH one-stage BC 112 and NIOSH 2-stage BC 212 samplers; Table2). This avoids the filter becoming blocked quickly by a large amount of relatively large particles.

**Table 4:** Comparison of commercially available filter air samplers

Sampler	Description	References	Flow Rate (L min^−1^)	Collection Efficiency d50 (µm)*	Sample period	Compatible with analysis method		
						Culture	Microscope	Biosensor/Molecular
IOM http://www.skcltd.com/index.php/air-sampling-pumps/9-uncategorised/457-iom-part-numbers-and-accessories-3	Filter (25-mm membrane or fibrous filter)	Aizenberg *et al.* (2000)	2	4		Yes (plating needed)	Yes (SEM and light mic possible)	Yes – requires processing steps
Button http://www.skcltd.com/index.php/air-sampling-pumps/9-uncategorised/204-button-sampler	Filter (25-mm membrane or fibrous filter)	Aizenberg *et al.* (2000)	4	4		Yes (plating needed)	Yes (SEM and light mic possible)	Yes – requires processing steps
DFU 1000	Dual dry filter unit (1 µm polyester filters)	Rosati & Drake-Richman (2009)	850	<1	continuous	Yes (plating needed)	Yes (processing steps needed)	Yes – compatible with hand held assays

* d50 is the aerodynamic diameter or size of particles above which 50% or more are collected and below which, less than 50% are collected.

## Non-capture or optical monitoring

This class of samplers, or sensors, operate not necessarily by capturing a sample but by using various optical sensors to analyse particles either suspended in the air, passed in an air-stream past a sensor (e.g WIBS; Table5), or entrained from air into a liquid that is then passed by a sensor (Day *et al.*, 2002). As with the VeroTect bio-detector and Biral Aspect samplers (shown in Table3 as they use virtual impaction) typically optical sensor-based samplers can operate continually by assessing the size of particles, their pigmentation (absorbance or reflectance) and/or fluorescence under certain excitationary wavebands from a source such as a laser or xenon lamp. Various analytical algorithms or more complex neural networks are used in ‘training’ studies to enable classification of unknown particles in an air sample but often cannot identify spores to a species level, only to a type (e.g. Day *et al.*, 2002). In the case of Light Detection and Ranging (LIDAR), a powerful laser is used in open air and the resulting reflectance from particles (and gases) may be used to infer presence of certain particles. Raman LIDAR can be used for detection of gaseous chemicals, whereas fluorescence LIDAR and depolarization LIDAR are used for determining shape and size of particles. For example, Noh *et al.* (2013) were able to use LIDAR to detect presence of airborne particles matching characteristics of pollen during the hay fever season.

**Table 5:** Comparison of non-capture air sampling (*see also the VeroTect bio-detector and Biral Aspect in Table3)

Sampler/Method	Description	References	Flow Rate (L min^−1^)	Collection Efficiency d50 (µm)*	Sample period	Analysis method
Any impinger or wet-cyclone	Particles entrained into liquid and analysed optically	Day *et al.* (2002)	Various	Depends on entrainment method	continuous	Particles entrained into a liquid pass by flow cytometry, an optical sensor that can classify particles according to size, scatter and pigmentation
wide issue bioaerosol spectrometer (WIBS)	optical particle counter with a dual wavelength fluorescence spectrometer	Gabey *et al.* (2013); Kaye *et al.* (2005)	<1 (i.e. maximum particle detection rate of 100 Hz (which equates to 40,000 particles /litre)	0.8–20	continuous	Various optical characteristics of individual particles in a narrow air-stream are recorded such as, particle size and symmetry, forward- and side-light scatter, fluorescence and absorbance and used to classify particles. Tryptophan fluorescence is included by excitation at 280 nm, while an illumination pulse at 370 nm is applied to excite NADH fluorescence. Resulting fluorescence in the wave bands 310–400 nm and 400–600 nm is measured.
BioLaz http://www.pmeasuring.com/particleCounter/microbialAirSamplers/BioLaz	Real-time biological particle detector by particle fluorescence		3.6	0.5–50	continuous	405 nm laser excites particles. Fluorescence from biological particles is detected and compared against light scattering caused by all particles to classify biological particles.
Lidar	Non-capture (optical sensing method)	Noh *et al.* (2013)	N/A	ID range varies with method, generally <50 µm	continuous	Classification based on particle size, shape and pigmentation but relatively imprecise with respect to species of biological particle.

* d50 is the aerodynamic diameter or size of particles above which 50% or more are collected and below which, less than 50% are collected.

## Sampler locations: field, rooftop and mobile platforms

The selection of a static sampler's location and height are important aspects of studying plant epidemiology and airborne disease dynamics. Sampler installation on a rooftop (10–30 m above ground) is recommended for monitoring dynamics in outdoor aerobiology (e.g. pollen) with a regional interest, as it is less dominated by localised effects of inoculum or air pollution (Khattab & Levetin, 2008). Fixed installation at ground level (0.5–1.5 m) is most often applied in sampling fungal spores that will be representative of airborne spore concentrations from localised sources or for determining dispersal gradients (Lacey & Venette, 1995). However, optimal sampler location and height is largely determined by the information required and should consider several factors, including the source and aerodynamics of the target bioaerosols, the surrounding vegetation or physical barriers, the requirements of the sampler, and meteorological conditions that affect release, dispersal and deposition of fungal spores in both vertical and horizontal gradients (Khattab & Levetin, 2008). Thresholds of spore concentrations that trigger actions need to be adjusted according to the sampler position with rooftop spore traps sampling air that is well-mixed from numerous potential sources within the region but also diluted with distance from the source (West *et al.*, 2008; West 2012). Within fields, the best sampling location is just above the crop canopy, where air is mixed from that escaping the crop with that arriving from external sources (Mahaffee, 2014). However, in this case, thresholds are difficult to interpret because a relatively high concentration of spores can be caused by a small release of spores immediately next to the spore trap and small local spore releases may be missed completely. Consequently, to improve reliability and confidence, it has been recommended to have three spore traps per field, e.g. for detection of *Botrytis squamosa* in onion fields (Carisse *et al.*, 2008), while in other cases (strawberry powdery mildew) only one spore trap was recommended to monitor a field (1400 m^2^)(Van der Heyden *et al.*, 2013). Only in a relatively few cases, have thresholds been identified that indicate a particular disease risk, e.g. 50 conidia m^−3^ d^−1^ of *Erysiphe necator* was identified in Quebec, but this was for a specific grape variety (cv Chancellor) (Carisse *et al.*, 2009b) and often the spore concentration or area under a spore curve needs to be combined with an infection model to indicate actual disease risk (Carisse *et al.*, 2012).

Major advances in computer systems, such as computational power and the miniaturisation of electronics, have now seen the emergence of new technologies like autonomous mobile vehicles, such as UAVs (Gonzalez *et al.*, 2011). Recent advances using this technology have allowed more cost effective applications of unmanned systems (e.g. ground robotics and aerial vehicles) emerge as viable remote sensing platforms. Savage *et al.* (2012) used computational models to simulate a number of dispersal events and showed that sampling strategies based on mobile platforms have a much greater probability of detecting airborne spores than strategies based on stationary traps, and that mobile trap strategies required a far lower number of traps to achieve a reasonable probability of detection. However, mobile sampling strategies using UAVs ideally should be in conjunction with stationary sampling installations as part of an area-wide sampling strategy to determine spore concentrations of pathogens or microbial contaminants (e.g. pollen), particularly the point of origin and dispersal rates, to fully realise the benefits of this emerging technology to predictive or risk support systems of aerobiology between neighbouring fields or growing regions (Aylor *et al.*, 2006, 2011).

The use of ground-based mobile vehicles, particularly in rural areas, confers the advantage of value adding to existing infrastructure the benefits from technical innovation in ground-based vehicle automation, such as the commercial auto-steer products using global positioning system (GPS). Even routine or irregular ground-based vehicle activities can offer valuable sampling platforms for aerobiology. Brown (1991) developed a mobile impaction sampler for monitoring pollen from motorways throughout England. The device used an intake slit (14 × 2 mm) which narrowed (14 × 0.5 mm) at the impaction point onto adhesive covered glass slide to obtain higher efficiencies than a stationary impaction sampler on which the mobile device was based (Brown & Jackson, 1978). Optimal speed for sampling was 80 km h^−1^, when the air intake is approximately 12 L min^−1^. Similarly, Limpert *et al.* (1999) describes the population dynamics of cereal mildews surveyed across Europe's motorways using Burkard's Jet Spore Samplers. Fixed installations, and mobile samplers mounted on a car roof, were used to capture spores onto Petri dishes containing barley leaf segments, which were exchanged after 100 km in each sampling region along a motorway. Real potential for up-scaling such simple mobile sampling systems will become apparent as more sophisticated technologies are applied that can automate capture, geo-reference collections, and be compatible to either rapid *in-situ* or downstream analysis (see next section). A recent example of this principle of sampling mobility with new technology was reported by Narayan *et al.* (2010) who implemented a tape rotation algorithm to control the position of a Hirst-type impaction drum to capture multiple samples on separate segments of the adhesive tape. Capture was controlled by a GPS location system integrated within the electronic hardware system to enable geo-location of the sampled spores to the ground vehicles' travel path. The same system was modified to reduce overall weight and improve aerodynamic requirements before being applied to a UAV platform (Gonzalez *et al.*, 2011).

The deployment of air samplers on mobile platforms has been reported in research studies in plant pathology over the last few decades (Gottwald & Tedders, 1985; Brown, 1991; Limpert *et al.*, 1999). These platforms offer novel ways in gathering important data in the field on pathogen spore dispersal and provide an extra dimension to ground-based approaches, allowing vertically layered sampling. However, most samplers detailed in this review were not developed with due consideration for mobile deployment, particularly in context of payload restrictions on aerial mobile vehicles. Therefore, early studies using mobile platforms describe modified samplers based on the principle of aerobiology capture of stationary samplers. Gottwald & Tedders (1985) describe a system using dual miniaturised Hirst-type impaction samplers (using an adhesive-coated melinex tape on a circular drum and a 1 mm air intake sampling at 12–14 L min^−1^) mounted on a remote-controlled biplane to the underside of its 2.44 m wingspan. The circular drum holding the spore collection surface was moved by a servo motor to new positions to capture pollen and spores over peach and pecan orchards as a vertical sampling method to compare with sampling of a stationary Burkard volumetric spore trap. Alternatively, Anderson *et al.* (1999) described a cyclone sampler type device applied to a UAV which used a custom ram-air-driven cyclone particle collector coupled to a multi-channel fluorimeter for air sampling experiments of airborne bacteria. The onboard fluorimeter (controlled by the ground station), provided real-time detection of pre-fluoresced particles in the atmosphere. While this example details sampling of unicellular microorganisms, it highlighted that the field of biosensor, or miniaturised DNA-based detection systems, is likely to offer great potential in the future for the broader field of aerobiology sampling that utilises mobile platforms.

More recent applications of UAV systems as mobile sampling platforms have been demonstrated (Schmale *et al.*, 2008; Gonzalez *et al.*, 2011; Aylor *et al.*, 2011). Schmale *et al.* (2008) utilised autonomous UAVs fitted with simple open and close impaction sampling devices to establish sampling strategies for high altitude aerobiological monitoring. Orbital sampling patterns around a single GPS waypoint exhibited high positional accuracy and sampling efficiencies. However, they reported that UAVs that operate independently of one another may experience significant sampling variations during the course of a flight, whereas the coordinated flight of two or more UAVs that enter, sample, and exit a spore plume at consistent times, sometimes referred to as swarming, would further realise the benefits of these platforms. This type of systematic sampling applied to aerobiology was subsequently demonstrated by Techy *et al.* (2010) using two synchronised autonomous UAVs to detect airborne sporangia of the potato late blight pathogen, *Phytophthora infestans*, in the lower atmosphere (25–45 m) above affected fields. Furthermore, Lin *et al.* (2013) and Schmale *et al.* (2012) also used impaction air samplers on a UAV to vertically sample air for *Fusarium* spores at heights up to 320 m and showed that below 100 m spores within this layer of air, closest to the Earth's surface, are likely to be deposited passively onto crops in still air at night. Particles higher than this can only descend by turbulence or by inclusion into rain (either by seeding ice or rain formation or by impaction by falling rain).

The diversity of UAV and affiliated systems are expanding rapidly to accommodate a range of sensors with increased mission capabilities from semi-autonomous to pre-programmed flight plans, wireless communication networks, accurate navigation systems and collision avoidance sensors (Anderson & Gaston, 2013). Autonomous UAV systems can potentially be flown with greater accuracy, significantly reduce pilot burden and operate more frequently than manned aircraft depending on the platform capability and performance limitations. Small low-cost UAVs such as multirotors (quadcopters, octacopters etc.) allow discontinuous trajectories such as hovering capabilities but may have payload, speed, power, flight time and endurance limitations (Hardin & Jensen, 2011), and any addition of an on-board air sampler must consider downward draft from rotor activity. Larger unmanned platforms (e.g. fixed-wing) have increased payload capacity or diversity of on-board sensors and equipment can offer greater potential for long-range high endurance applications such as large scale aerobiological sampling strategies (Schmale *et al.*, 2008; Gonzalez *et al.*, 2011). However, complexity and capability requires additional specialist skills for the operator, greater safety implications and compliance to strict operating guidelines with governing aviation safety regulations (Hardin & Jensen, 2011).

## Automation of air sampling

The Hirst spore trap (Hirst, 1952) was considered to be an automatic spore trap. In a sense, that was correct, as it collected a sample over an extended time period without any intervention. However, in this section, we will consider devices and methods that automate not only detection, but analysis and reporting of the result. Great advancements have been made in automatic detection of airborne particles, particularly in the fields of bio-warfare and homeland security. This is a very rapidly advancing area and numerous devices have been developed – many capable of detecting a certain class of microbe, e.g. all bacteria, or all fungal spores of a certain size, in near-real-time. Many use wet-cyclones, virtual impactors, impactors or filters, or combinations of these to collect a sample to which is applied a diagnostic test. The majority capture or transfer the sample into a liquid format, which is more easily moved and dispensed onto a diagnostic but only a few systems have been developed that can detect and quantify an individual species in near-real-time. For example the Biotrace Biological Detection System uses a wet-walled cyclone with high sample collection efficiency and flow rate of up to 750 L min^−1^. The sample passes an ATP detector in a continuous flow with microbial ATP driving bioluminescence, for rapid detection of any viable biological material. In response to attacks using letters containing Anthrax spores in 2001, which caused the deaths of five people, the USA developed a non-portable Autonomous Pathogen Detection System. This was a file-cabinet-sized device that sampled air, entraining particles into liquid for flow cytometry, sample preparation, real-time PCR and reporting of results. Up to 100 biological agents could be detected per sample. However, later versions used bead-capture immunoassays and can run continuously for 24 h. This was augmented by the ‘Biobriefcase’ (patent US20060281101A1 and additional associated patents), which is a more compact system for sampling airborne particles using an immunoassay section and/or a nucleic acid assay section within a housing. The sample can be sub-divided to allow testing for different agents by these different methods. In 2010, patent US007799567B1 described a system using virtual impactors to concentrate airborne particles and impact them onto a bed of beads (which reduces particle resuspension or ‘bounce’). A sub-sample of the beads is automatically transferred into an automated analytical system. However, most, if not all of these are too expensive for applications in practical plant pathology. Instead, a recent development of the ‘SYield’ project offers potential of automated detection of spores of individual species after a set sampling period. So far the system has been tested only for detection of *Sclerotinia sclerotiorum* (West *et al.*, 2013), which is sporadic in the UK and a serious problem in other locations. The ‘SYield’ system (West *et al.*, 2013) uses a miniature virtual impactor (MVI; Table3) to trap airborne particles, which are then incubated in a semi-selective liquid growth medium. After incubation, an assay for oxalic acid is made using a biosensor. Each day, results from the incubated samples are transmitted wirelessly to a server, along with hourly meteorological data collected from an integrated weather station. These results are processed to make a risk prediction, which is texted to the farmer. The system is intended to work as a network of sensors, which will reinforce risk alerts on a regional scale.

## Conclusions

As Jim Hirst wrote (Hirst, 1995), ‘It is usually essential to consider the environment and purpose of an investigation before deciding which [spore] trap to use and where to locate it’.

The evolution of spore traps has continued since the Hirst trap was developed, using the second air intake of a cascade impactor and sample collection changing from a waxed slide to a tape on a circular drum. Increasingly, samplers that sample into tubes or other vials are being used to make processing steps more user-friendly and even to facilitate automated testing of samples. In some cases, automation of sampling (Multivial cyclone) was then augmented by automated processing steps to apply samples to biosensors (MVI). Already, work is in process to automate application of immunological and isothermal DNA assays to detect spores with results sent wirelessly to avoid the delays that would occur if samples had to be sent to a laboratory.

The development of air samplers is likely to produce a step change in their use with much greater adoption by growers to support crop protection decisions. The same devices are likely to improve the information available for detection of allergens causing hay fever and asthma. Devices mounted on vehicles and UAVs are likely to be used increasingly both in research, to understand locations of sources and dispersal processes, and in practical risk alert and monitoring networks. Ground-based automated samplers and those designed to facilitate easy analytical steps by non-specialists are also likely to benefit from simpler, cheaper and more rapid diagnostic methods to add precision on disease control decisions at the field, farm and regional scales.

Laboratory-based methods may still prove to be cost effective if multiple target organisms can be detected cheaply. This is likely to remain the case for generic studies of the air-spora community, particularly for unknown fungi, bacteria and viruses or viroids in other particles, using methods such as terminal restriction fragment length polymorphism, denaturing gradient gel electrophoresis and next generation sequencing.

The thresholds of spore concentrations used to trigger disease control operations, require a great deal of consideration because the same concentration of spores detected could be caused by either a large, distant source (with spores diluted over a regional scale) or a relatively small but very local source of spores (i.e. with little dilution prior to being sampled). Ideally decisions should, therefore, be based on integration of results from a network of samplers, or use a sampler that is buffered from potentially close releases of spores by locating the spore trap well above the ground, such as on the roof of a tall building. For common plant pathogens it is possible to infer presence of airborne inoculum over a regional scale from a single air sampler located at rooftop height (West, 2012). Unfortunately, unless a particularly high volume air sampler is use, most types of air samplers cannot be used for biosecurity purposes to detect very rare influx of exotic spores (Jackson & Bayliss 2011).
